# Varicella zoster virus infection presenting as isolated diplopia: a case report

**DOI:** 10.1186/1471-2334-13-138

**Published:** 2013-03-15

**Authors:** Raffaella Pisapia, Alessia Rianda, Andrea Mariano, Angela Testa, Simonetta Galgani, Laura Vincenzi, Amina Abdeddaim, Alessandra Oliva, Pasquale Narciso

**Affiliations:** 1 Clinical Department, First Division, National Institute for Infectious Diseases “Lazzaro Spallanzani”, Via Portuense 292, 00149, Rome, Italy; 2 Neuroscience Department, “San Camillo Forlanini” hospital, Rome, Italy

**Keywords:** Varicella zoster virus, Cranial nerve palsy, Molecular diagnosis, Clinical awareness

## Abstract

**Background:**

Involvement of trochlear nerve during Varicella Zoster Virus (VZV) Infection has been rarely described, and always in association with skin rash.

**Case presentation:**

We describe the case of a patient with VZV infection presenting as isolated diplopia due to fourth cranial nerve palsy. The diagnosis has been obtained through the application of a standardized molecular diagnostic panel, and diplopia resolved after specific antiviral and corticosteroid therapy.

**Conclusion:**

This case evidences that clinicians should be aware of atypical VZV infection, even in the absence of the typical skin rash.

## Background

Varicella Zoster Virus (VZV) often causes central nervous system complications either during primary infection (chickenpox) and during reactivations. Neurological involvement includes a wide spectrum of diseases, from benign acute aseptic meningitis to severe manifestations, with meningitis and encephalitis, acute cerebellar ataxia, Guillain-Barré syndrome, Reye’s syndrome, myelitis, optic neuritis more frequently reported in case of primary varicella infection, and vasculopathy, focal motor weakness, Ramsay-Hunt syndrome, post-herpetic neuralgia and cranial nerves palsy more frequently reported in case of reactivation [1,2].

The etiological diagnosis of these cases is often challenging for the clinicians, especially when these manifestations occur without the typical vesicular rash [1,2].

Here we describe the case of an adult patient, immune-competent and without underlying diseases, with fourth nerve palsy, presenting diplopia as isolated symptom of central nervous system involvement during VZV infection without rash.

## Methods

A specific search on PubMed about the occurrence of nerve palsy during VZV infection has been performed, using the terms “Varicella Zoster Virus Infection”, “Herpes Zoster”, “Shingles” and “Chickenpox” as major topics, each matched with “nerve palsy”, or “neurological complications”. The bibliography of relevant articles have been checked to identify other significant papers.

## Case presentation

A 33-year-old man was referred to the emergency department and admitted (day 1) to a general hospital for acute onset of isolated diplopia six hours prior. Seven days prior to admission, he developed an unspecific syndrome, characterized by mild headache, generalised myalgia, vomiting and nausea without fever. Neurological examination was unremarkable except for vertical and cross diplopia at left eye suggesting an isolated fourth nerve palsy. Ocular examination including visual field and fundoscope assessment were normal and no pathological findings were observed on Brain CT Scan.

Cerebro-spinal fluid (CSF) analysis performed in the same day showed pleocytosis (white blood cells count of 969/mm^3^ with normal value < 2/mm^3^, 80% lymphocytes), proteins 188 mg/dl (normal value 8–32 mg/dl) and glucose 51 mg/dl (normal value 40–70 mg/dl), with a blood glucose of 111 mg/dl (normal value 70-110 mmg/dl). Gram stain was negative. The day after, in the suspicion of a viral encephalitis, antiviral therapy with intravenous acyclovir was started at the dose of 10 mg/kg every 8 hours and the patient was referred to our Infectious Diseases hospital.

At admission in our hospital (day 2) his clinical condition was unchanged. We continued antiviral therapy and added anti microbial therapy with ceftriaxone 2 grams twice a day, and ampicillin 2 grams 6 times a day.

Diplopia persisted despite treatment, so on day 4 a second lumbar puncture was performed. The CSF analysis showed 200 WBC/mm^3^ (normal value < 2/mm^3^), protein 200 mg/dl (normal value 8–32 mg/dl) and glucose 56 mg/dl (normal value 40–70 mg/dl), with a blood glucose of 94 mg/dl (normal value 70-110 mmg/dl). Routine tests were normal. Magnetic Resonance Imaging (MRI) of the brain showed right sphenoid sinusitis only. Chest X-ray was normal.

The agents most frequently responsible of meningo-encephalitis were investigated: serological tests for VZV, cytomegalovirus, herpes simplex virus 1–2, Epstein Barr virus, measles virus, mumps virus, rubella virus, adenovirus, enterovirus, influenza and parainfluenza virus, reovirus, respiratory syncytial virus, tick-borne encephalitis agents, West Nile virus, Toscana virus were all negative. A PCR performed on pharyngeal and rectal swab resulted negative for enterovirus, adenovirus and respiratory viruses. Additional examinations included serology for Brucella, Listeria, Treponema, Coxiella, Mycoplasma, Chlamydia, Borrelia, Leptospira, Criptococcus antigen, Quantiferon, HIV antigen and antibody test, Legionella and pneumococcal urinary antigen, autoantibodies. All these tests resulted negative.

A set of quantitative real-time PCR performed on CSF was negative for herpes simplex virus 1–2, human herpes virus 6, cytomegalovirus, Epstein Barr virus, enteroviruses, measles virus, mumps virus and M. tubercolosis, while a quantitative real-time PCR (HSV-VZV R-Gene Quantification Test, Biomerieux®) resulted positive for VZV-DNA, with a viral load of 30360 copies/ml.

On day 8 after initial admission, before a definitive diagnosis was established, corticosteroid therapy with oral prednisone was started at the initial dose of 50 mg once daily and then at tapering dosages over a 40 days course, as suggested by neurologist consultant. Partial improvement of diplopia was observed after three days. Antibiotics were stopped on day 12, while acyclovir was continued up to day 14. The diplopia progressively improved, and the patient was discharged on day 23. Forty-five days later, the patient reported the complete disappearance of diplopia. Full resolution of oculo-motor impairment was evidenced by an Hess-Lancaster test performed 3 months later.

## Discussion

The impairment of central nervous system is not rare during VZV infection. Indeed, VZV is now recognized as one of the main cause of adult encephalitis: in recent series on encephalitis from Europe, VZV has been identified as the second most common etiology, remaining Herpes Simplex the first one [3,4]. Diagnosis of VZV is usually suggested by the presence of the characteristic skin rash. However, all neurological diseases following VZV infection can occur in absence of the typical vesicles, making the clinical suspicion of VZV challenging for the clinician [1,2].

Neurological complications during VZV infection include isolated cranial nerve palsies. The third and the sixth nerve are most frequently involved as well as peripheral facial palsy (Ramsay Hunt Syndrome), while the involvement of the fourth nerve is rarely described, and always in association with shingles or during herpes zoster ophthalmicus [5,6].

Conversely, our patient presented with isolated diplopia in absence of the typical rash and of any other signs of neurological impairment, except for a mild headache a week before. The diagnosis has been virologically demonstrated by the detection of VZV-DNA in the CSF. It has been possible through the application of extensive molecular test panel, based on PCR assays, on CSF. This panel includes the most common infectious causes of neurological symptoms.

The diagnostic approach for neurological complications should be supported by PCR amplification of VZV DNA in CSF and the search of intra-thecal IgG antibody production, or specific IgM in the CSF. The detection of intra-thecal IgG showed to be useful in the improvement of VZV-related vasculopathy and myelopathy diagnosis, when VZV is not detectable [7]. In our case the diagnosis was sustained by PCR, and intrathecal anti-VZV antibodies were not investigated.

Antiviral therapy was promptly started, and corticosteroids were added at day 10. Some experiences suggested the possible role of short-term corticosteroid therapy, in adjunction to antiviral therapy, to reduce inflammation [7]. Indeed, the pathogenesis of nerve palsy during VZV infection is not fully understood. It may involve (i) a direct cytopathic damage by retrograde spread of the virus; (ii) a vasculopathy caused by active viral replication; or (iii) a circumscribed orbital myositis. Corticosteroids have been proposed for primary VZV encephalitis and in immunocompetent patients with severe VZV encephalitis and vasculopathy, but there are no definitive data supporting their use [8].

## Conclusion

This case shows that clinicians should be aware of the role of VZV in neurological symptoms, even if the typical skin rash is not present. Indeed, if the role of VZV is unrecognized, appropriate antiviral medications may be discontinued early, once HSV is ruled out. The application of a standardized panel on CSF, including molecular tests for more common agents causing neurological impairment, could be very useful.

## Consent

Written informed consent has been obtained from the patient for publication of this Case report. A copy of the written consent is available for check by the Editor of BMC Infectious Diseases, if needed.

## Competing interests

The authors declare that they have no competing interests.

## Authors’ contributions

RP collected the clinical data and drafted of the manuscript. AR, AM, LV, AA, and AO managed the patient, and supported RP in the collection of clinical data and drafting of the manuscript, AT performed the literature search and support RP in the drafting of the manuscript, SG contributed to the clinical and therapeutic management from a neurological point-of-view, and revised the neurological details of the manuscript, PN supervised the clinical case interpretation, participated in the coordination and concept of the manuscript, and helped with the draft of the manuscript. All authors read and approved the manuscript.

## Pre-publication history

The pre-publication history for this paper can be accessed here:

http://www.biomedcentral.com/1471-2334/13/138/prepub
